# Knowledge, Attitude, Practices, and Associated Factor towards Hepatitis B Virus Infection among Health Care Professionals at Tibebe Ghion Specialized Hospital, Bahir Dar, Northwest Ethiopia, 2021: A Cross Sectional Study

**DOI:** 10.1155/2022/3726423

**Published:** 2022-05-05

**Authors:** Debaka Belete, Dagnaneh Wondale, Teklehaimanot Kiros, Biruk Demissie

**Affiliations:** ^1^University of Gondar, College of Medicine and Health Sciences, School of Biomedical and Laboratory Sciences, Department of Medical Microbiology, Gondar, Ethiopia; ^2^Deberetabore University, College of Health Sciences and School of Medicine Department of Medical Laboratory, Deberetabore, Ethiopia; ^3^Deberetabore University, College of Health Sciences and School of Medicine Department of Public Health, Deberetabore, Ethiopia

## Abstract

**Background:**

Hepatitis B virus (HBV) infection is the major infectious hazard for health care personnel. The global prevalence of HBV infection is highly heterogeneous, and the highest prevalence (6.2 and 6.1%) is among the World Health Organization Western Pacific and World Health Organization African regions, respectively. The pooled prevalence of HBV in Ethiopia among health workers was accounted for 5%. The prevalence rate of HBV in health care workers is about 2–10 times higher than the general population in the world. There for, the main aim of this study was to assess the knowledge, attitude and practice, and associated factors towards hepatitis B virus (HBV) infection among health care professionals at Tibebe Ghion Specialized Hospital, Bahir Dar, Northwest Ethiopia, 2021.

**Method:**

An institutional-based cross-sectional study design was at Tibebe Ghion Specialized Hospital, Bahir Dar, in 2021, and a systematic random sampling technique was used from different professionals, and the separate sample was taken independently from each. A pretested structured questionnaire was constructed and collects data then analyzed by using SPSS version 23.

**Result:**

A total of 422 health care workers having different professions have participated in this study. 243 (57.6%) of the study subjects were males. The average correctly answered knowledge, attitude, and practice questions were 65.6%, 40.3%, and 34.8, respectively. Multivariable logistic regression analysis showed that being nurse professionals (*AOR* = 0.17 (0.07, 0.38), *P* < 0.001), midwives (*AOR* = 0.19 (0.07, 0.5), *P* = 0.001), and work experience (*AOR* = 2.37 (1.38, 4.02), *P* = 0.002) were associated with knowledge levels. Being degree holders (*AOR* = 2.49 (1.23, 5.02), *P* = 0.01) and specialists (*AOR* = 9.78 (2.69, 35.5), *P* = 0.001) were associated with attitude levels. Being medical laboratories (*AOR* = 17.42 (5.02, 60.5), *P* ≤ 0.001) and pharmacy professionals (*AOR* = 11.2 (4.02, 31.42), *P* ≤ 0.001) were associated with practice levels. *Conclusion and Recommendation*. Based on the current study, most of the health care professionals in this study area have poor knowledge, negative attitude, and malpractice towards HBV infection. Therefore, continual professional training programs on HBV infection include increased vaccination coverage rate and postexposure prophylaxis of heath care workers especially for highly exposed professionals.

## 1. Introduction

Hepatitis B virus (HBV) is an established chronic infection, especially in those infected as infants; it is a major factor in the eventual development of liver disease and hepatocellular carcinoma in those individuals [1]. It is a highly infectious virus in the blood of both symptomatic and asymptomatic patients; chronically infected individuals pose a serious threat to all health care workers, and immunization of such individuals is generally required [2]. Hepatitis B virus (HBV) infection is the major infectious hazard for health care personnel. Health care workers (HCWs) are at high risk of HBV infection in health care settings. Hepatitis infection is one of the major public health problems globally and is the tenth leading cause of death [3].

The transmission of infectious HBV is present in all body fluids of an infected individual. Therefore, blood, semen, saliva, and mother's milk, for example, serve as sources of infection, and also it is spread by needle stick injury [2, 4, 5]. The risk for acquiring HBV infection from occupational exposures is dependent on the frequency of percutaneous and per mucosal exposures to blood or body fluids containing blood [6, 7]. WHO has estimated that, in 2000, injections with contaminated syringes caused 21 million hepatitis B viruses (HBV) infections [6]. Epidemiological studies indicate that a person who experiences one needle-stick injury from a needle used on an infected source patient has a risk of 30% to become infected with HBV [7].

The pathogenesis of HBV is that fully differentiated hepatocytes are the primary cell type infected by HBV. The disease has both acute and chronic phases, with the acute phase being a new infection. After six months of persistence, the acute phase often results in chronic infection, which lasts a lifetime [8]. HBV is of medical and public health importance, not only as the cause of acute liver disease but also as the cause of chronic, persistent infections that can result in the eventual death of infected individuals from cirrhosis and liver cancer [2]. Hepatitis B virus (HBV) infection is a lifelong dynamic disease that can be controlled with treatment but cannot yet be cured [9].

The diagnosis of hepatitis is made on clinical grounds, coupled with biochemical tests that evaluate liver damage. Elevations of aminotransferases, bilirubin, and prothrombin time all contribute to the initial evaluation of hepatitis. In addition, identification of the presence or absence of specific antiviral antibodies and viral antigens permits differentiating between acute and chronic HBV infections [2]. For the prevention of HBV, the purpose of controlling the spread of HBV infection is to prevent cases of acute hepatitis [2]. A vaccine against hepatitis B has been available since 1982 with the efficacy 85-90% in preventing infection [10]. Hepatitis B infection transmission chain can be interrupted through vaccination, use safe precautions while handling infectious material, and proper sterilization of medical equipment [3]. Individuals with HBV infection should receive anti-HBV treatment if they have elevated ALT level and elevated HBV DNA level [9]. Patients with HBV infection who should be treated include those with chronic hepatitis, those with cirrhosis, and those with hepatocellular carcinoma [9]. The best way to prevent hepatitis B is by getting vaccinated [11]. The complete vaccine series induces protective antibody levels in more than 95% of infants, children, and young adults [7].

Throughout the world, millions of health care professionals work in health institutions, and it is estimated that 600,000 to 800,000 experiences cut and puncture injuries occurring among them per year, of which approximately 50% are not registered. The annual proportion of health care workers exposed to blood-borne pathogens was 5.9% for hepatitis B [8]. The study was conducted in Ethiopia, for instance, showed that 7.3% of health care workers (HCWs) were infected with HBV while only 0.9% of non-HCWs [7]; however, this study was not assess the KAP of HCWs.

For this reason, the infection of HBV among health care professionals is important public health problems, but to the best of our knowledge, there is no study conducted in this area; hence, conducting this study and address, this issue may fill the existing gap. So the main objective of this study is intended to assess the KAP towards HBV infection among health care professionals at Tibebe Ghion Specialized Hospital, Bahir Dar, Northwest Ethiopia, 2021.

## 2. Methods and Materials

### 2.1. Study Design, Area, and Setting

Institutional-based cross sectional study design was conducted at Tibebe Ghion Specialized Hospital (TGSH), Bahir Dar, Northwest Ethiopia, from January 1 to February 30/2020. Tibebe Ghion Specialized Hospital is one of the specialized hospital located in Amhara region at Bahir Dar city, and it gives service for the community around Bahir Dar and the region at large.

Bahir Dar is the capital city of Amhara regional state in Northwest Ethiopia. The city has 3 governmental hospitals, 10 health centers, and 12 health posts, and also, there are many private hospitals and clinics as well as different institutions in the city. From those health institutions, Tibebe Ghion Specialized Hospital is found at the south east direction of the Bahir Dar city. During the study period in Tibebe Ghion Specialized Hospital, there were 536 health care professionals. These are 118 medical doctors, 266 nurses, 61 midwives, 41 pharmacy professionals, 26 medical laboratory professionals, 8 anesthetists, 6 radiographers, 6 psychiatrists, 2 dentists, and 2 ophthalmologist.

### 2.2. Study Population, Sample Size, and Sampling Technique

All health care professionals include medical doctors, medical laboratory, nurses, midwives, pharmacist, dentist, psychiatrist, ophthalmologist, radiologist, and anesthetist at Tibebe Ghion specialize hospital. The sample size was determined using 50% prevalence because we have not got the literature about assessment of knowledge, attitude, and practice towards hepatitis B virus infection among health care professionals in Ethiopia. The sample size was determined using the following formula: *N* = *z* (1-*α*)/2)^2^*p* (1*p*)/*d*^2^, where *N* is the minimum sample size, *p* is an estimated prevalence rate for the population, *d* is the margin of sample tolerated, and *z* (1 − *α*)^2^ is the standard normal variable at (1 − *α*)% confidence, taken as 5% and 95% confidence interval. Accordingly, the sample size calculated was 422.

A stratified sampling technique was used to make a strata for different professionals, and a separate sample was taken by proportion to allocate the professionals based on their numbers independently from each stratum by systematic random sampling technique to select study subjects among health care professionals who are working at Tibebe Ghion Specialized Hospital from January 1 to February 30/2020.

### 2.3. Operational Definitions

- Knowledge

1. *Good knowledge*: if the respondents were able to answer 70% or more of knowledge items correctly [7]
2. *Poor knowledge*: if the respondents answered less than 70% of knowledge items [7]

(ii) Attitude

1. *Positive attitude*: if the respondents were are able to give the correct answer for 70% or more of attitude items
2. *Negative attitude*: if the respondents answered less than 70% of attitude items [7]

(iii) Practice

1. *Good practice*: when the study participants were at least able to answer 70% or more practice items correctly [7]
2. *Poor practice*: when the participants were unable to answer 70% of practice items correctly [7]

### 2.4. Data Collection Tool, Procedure, and Data Quality Assurance

Data were collected by using a structured questionnaire. The questionnaire was containing written consents, socio-demographic variables, knowledge, attitude, and practice questions towards hepatitis B infection prevention which were developed by adapting from different peer-reviewed literature.

For ensuring data quality, 5% of the questionnaire was pretested before the actual data collection process. In addition to this, the semistructured questionnaire was prepared in the English version and translated in the local language (Amharic version) and then transcribed back to English to maintain its consistency. Moreover, adequate training was given for data collectors and supervisors. Generally, the process of the study was supervised daily so that any incompletely filled questionnaires.

### 2.5. Data Processing and Statistical Analysis

The collected data was checked for its completeness, and then, data was entered into SPSS version 23. Statistical analysis for descriptive statistics of the variables was computed to summarize the data. For categorical variables, frequencies and percentages were computed while for the continuous variable mean and the standard deviation were calculated. For the associated risk factor analysis of independent variables with the outcomes, the bivariable and multivariable logistic regression model was fitted. Odds ratios (OR) with 95% confidence intervals (95% CI) were calculated. All variables with *P* value < 0.25 (to control the effect of confounding) in the bivariate analysis were included in the multivariate logistic regression model for risk factor analysis so that adjusted odds ratio (AOR) with 95% confidence intervals was calculated. However, the model fitness of the final binary logistic regression was tested by using Hosmer and Lemeshow test at a *P* value > 0.05. In all cases, *P* value ≤ 0.05 was taken as a statistically significant association. Finally, the findings were represented with texts and tables

### 2.6. Ethical Clearance

The study was approved by the Debre Tabor University, College of Health Sciences and Department of Medical Laboratory Science, Research and Ethical Review Committee (Permission letter's reference number: CHS/224/2012 in Ethiopian calendar, Date 3/2/2012 E.C). Permission letter was obtained and sent to all concerned bodies, and the study was secured at all levels. The purpose, the potential benefits, and the possible risks associated with participation in this research work have been cleared so that participants were decided either to proceed or withdraw from the entire study. Also, study participants were informed both verbally and written so that a written consent form was given after a brief explanation of the study. All results were kept confidential, and the process was solely made through coding to maintain individuals' privacy concerns. However, the participants were given the full right to withdraw at any time from participating in the research process.

## 3. Result

### 3.1. Sociodemographic Characteristics

A total of 422 health care workers having different professions were participated in the study at Tibebe Ghion Specialized Hospital. Out of the total study participant, 243 (57.6%) were males. Of the study participants, 308 (73%) belong to the age group 26 to 33 with mean age of 29 with a minimum and a maximum age of 20 and 38, respectively. The majority of the study participants were nurses (49.8%) of the total population. Majority of the study participants were married (54%). Majority of the study participants were first degree (64.2%). 324 (76.8%) of the respondents had greater than 2 years' work experience (Table 1).

### 3.2. Assessment of Knowledge Level towards HBV

Most of the study participants had adequate knowledge on HBV infection and its mode of transmission. 359 (85.1%) knew that HBV can cause liver cancer. 350 (82.9%) of the participants knew that HBV vaccine can prevent HBV infection, but relatively, a low proportion of 174 (41.2%) knew that HBV has a postexposure prophylaxis. Among the professions, medical doctors (91.4%) were more knowledgeable followed by pharmacist (84.4%). The average knowledge level of the professionals was 65.6% (Tables 2 and 3).

### 3.3. Assessment of Attitude Level towards HBV

Of 98 (23.2%), the study participants had no concern being infected with HBV, and of 143 (33.6%), participants had a belief that changing glove is a waste of time. In contrast of 274 (64.9%), the participants believed that HBV vaccine is safe and effective. Of 307 (72.7%), study participants believed that following infection control guideline would protect them from HBV infection, but only 31.3% participants believed that postexposure prophylaxis could prevent from HBV infection. The average positive attitude levels of the professionals were 40.3% (Tables 4 and 5).

### 3.4. Assessment of Practice Level towards HBV

Of the total, 158 (37.4%) study participants were screened for HBV. However, the majority of 255 (60.4%) health care workers had vaccinated against HBV. About 410 (97.2%) of the respondents did wear glove, but 191 (45.3%) of the respondents were change glove for each patient. Of 192 (45.5%), the participants had a needle stick injury. Of 274 (64.9%), the participants had a needle stick injury. Of 274 (64.9%), the participants disposed sharps properly. The average good practice levels of the professionals were 34.8% (Tables 6 and 7).

**Figure 1:**
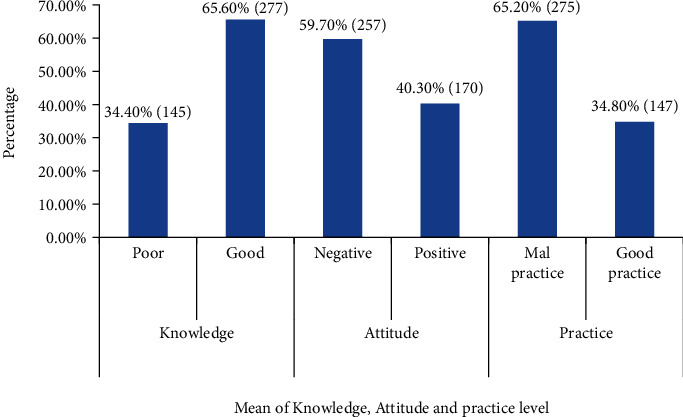
Mean KAP levels of the respondents about HBV early detection, transmission, and prevention.

Of the total, 277 (65.6%) study participants had good knowledge for HBV early detection, transmission, and prevention. However, the majority of 257 (59.7%) health care workers had negative attitude against HBV transmission and preventions. About 275 (65.2%) of the respondents had malpractice, but 147 (34.4%) of the respondents had good practice. The average good knowledge, positive attitude, and good practice levels of the study participants were 65.6% (277), 40.30 (170), and 34.8% (147), respectively (Figure 1).

### 3.5. Factors Associated with Knowledge Level towards HBV Infection

The result of binary logistic regression analysis showed that 2 of the 6 variables did not show a significant association with good knowledge at the 5% level of significance. From the factors, age and marital status were not significant. Sex, profession, educational status, and work experience were significant.

In a binary logistic regression analysis, being males was 2 times more likely to have a good knowledge than females (COR = 2.03, 95% CI (1.35, 3.05), *P* = 0.001). Being anesthetist had 98% less likely to have a good knowledge on HBV than pharmacy professionals (COR = 0.02, 95% CI (0.002, 0.18), *P* = 0.001). Being midwives had 89% have a good knowledge on HBV than nurse professionals (COR = 0.11, 95% CI (0.04, 0.28), *P* < 0.001). Similarly, being nurse professionals had 89% times less likely to have better knowledge on HBV than medical laboratories (COR = 0.11, 95% CI (0.05, 0.24)). In the multivariable logistic regression analysis, three variables had shown overall significant effect on good knowledge at 0.2 level of significance. These are professions, educational status, and work experience. Among the professions, being anesthetist had 98% times less likely to have good knowledge than pharmacy professionals (AOR = 0.02, 95% CI (0.002, 0.23), *P* = 0.001). Being midwife professionals had 81% times less likely to have good knowledge than nurse professionals (AOR = 0.19, 95% CI (0.07, 0.5), *P* = 0.001), and being nurse professionals had 83% times less likely to have good knowledge on HBV than medical laboratories (AOR = 0.17, 95% CI (0.07, 0.38), *P* < 0.001). Being health care professionals with greater than 2 years of work experience had 2.4 times more likely to have good knowledge on HBV than less than 2 years of work experience (AOR = 2.37, 95% CI (1.38, 4.07), *P* = 0.002) (Table 8).

### 3.6. Factors Associated with Attitude Level towards HBV Infection

The result of binary logistic regression analysis showed that 3 of the 6 variables did not show significant association with a positive attitude at a 5% level of significance. From the factors, age, marital status, and work experience were not significant. Sex, profession, and educational status were significant.

In binary logistic regression analysis, being males was 72.6% less likely to have a positive attitude on HBV than females (COR = 2.11, 95% CI (1.42, 3.18), *P* ≤ 0.001). Being nurse professionals had 69% times less likely to have a positive attitude on HBV than medical laboratory professionals (COR = 0.31, 95% CI (0.19, 0.51), *P* ≤ 0.001). Similarly, being midwife professionals had 64% times less likely to have a positive attitude on HBV than nurse professionals (COR = 0.36, 95% CI (0.17, 0.75), *P* = 0.006). Regarding the educational status, being degree holders had 2.5 times more likely to have a positive attitude on HBV than diplomas (COR = 2.5, 95% CI (0.126, 4.66), *P* = 0.006). Being MSc had 3.1 times more likely to have a positive attitude on HBV than degree holders (COR = 3.1, 95% CI (1.44, 6.85), *P* = 0.004). Similarly, being specialists had 15.6 times more likely to have a positive attitude on HBV than MSc (COR = 15.6, 95% CI (4.99, 48.78), *P* ≤ 0.001). In the multivariable logistic regression analysis, one variable had shown an overall significant effect on a positive attitude; this is educational status. Being degree holders had 2.49 times more likely to have a positive attitude on HBV than diplomas (AOR = 2.49, 95% CI (1.23, 5.02), *P* = 0.01). Being MSc had 3.97 times more likely to have a positive attitude on HBV than degree holders (AOR = 3.97, 95% CI (1.69, 9.28), *P* = 0.001). Similarly, being specialists had 9.8 times more likely to have a positive attitude on HBV than MSc (AOR = 9.78, 95% CI (2.69, 35.5), *P* = 0.001) (Table 9).

### 3.7. Factors Associated with Practice Level towards HBV Infection

The result of binary logistic regression analysis showed that 3 of the 6 variables did not show significant association with good practice at a 5% level of significance. From the factors, age, marital status, and work experience were not significant. Sex, profession, and educational status were significant. In binary logistic regression analysis, being males was 1.96 times more likely to have good practice than females (COR = 1.96, 95% CI (1.29, 2.99), *P* = 0.002). Related to professions, being medical laboratories had 4.9 times more likely to have good practice than medical doctors (COR = 4.9, 95% CI (1.71, 14.01), *P* = 0.003). Similarly, being pharmacy professionals had 4.62 times more likely to have good practice than midwives (COR = 4.62, 95% CI (1.95, 10.97), *P* = 0.001). Regarding educational status, being degree holders was 2.2 times more likely to have good practice than diplomas (COR = 2.2, 95% CI 2.2 (1.12, 4.32), *P* = 0.02). Being MSc was 4.06 times more likely to have good practice than degree holders (COR = 4.06, 95% CI (1.81, 9.12), *P* ≤ 0.001). Similarly, being specialist was 10.13 times more likely to have good practice than MSc (COR = 10.13, 95% CI (3.57, 28.69), *P* ≤ 0.001). In the multivariable logistic regression analysis, two variables had shown overall significant effect on good practice and are professions and educational status. Among the professions, being medical laboratories had 17.42 times more likely to have good practice than medical doctors (AOR = 17.42, 95% CI (5.02, 60.5), *P* ≤ 0.001). Similarly, being pharmacy professionals had 11.2 times more likely to have good practice than midwives (AOR = 11.2, 95% CI (4.02, 31.42), *P* ≤ 0.001). Regarding the educational status, being degree holders had 3.4 times more likely to have good practice than diplomas (AOR = 3.4, 95% CI (1.58, 7.35), *P* = 0.002). Being MSc had 5.3 times more likely to have good practice than degree holders (AOR = 5.3, 95% CI (2.12, 13.11), *P* < 0.001). Similarly, being specialist was 31.4 times more likely to have good practice than MSc (AOR = 31.4, 95% CI (8.59, 114.5), *P* ≤ 0.001) (Table 10).

## 4. Discussion

This study showed that doctors (91.4%) have answered correctly knowledge questions followed by pharmacists (84.4%), psychiatrists (83.3), and laboratory professionals (80%), respectively. Most of the study participants had adequate knowledge of HBV infection and its mode of transmission. Of 359 (85.1%) knew that HBV can cause liver cancer. Of 350 (82.9%) of the participants know that HBV vaccine can prevent HBV infection, but relatively, a low proportion of 174 (41.2%) know that HBV has postexposure prophylaxis. Based on our study majourty of study participants, 279 (65.6%) had good knowelege towards HBV infection. This finding was inconsistance with the study done in Mogadishu Somalia (80.9%) [12], Northern Nigeria (76.9%) [6], and khartoum, Sudan (70.0%) [13], respectively. This is maybe the difference in the educational status of the study population. However, it is higher than the study that was conducted in Vietnam 59.5% [14] and in Lagos State, South-Western Nigeria; among all the doctors and nurses in the health care facility, 56.7% [15] had an average level of knowledge. This is may be that this study was done at specialized hospitals which have better educational status than the others.

This study showed that professions, educational status, and work experience were significantly associated with a good knowledge level; 18. This finding was consistent with the study done in Bamenda Health District, NWR, Cameroon; education and different HCW professions were associated with knowledge level. This may be the result of study participant similarity. 19. In this study, all health care professions in different categories were incorporated in cotrast Saad Abul-Ella hospitals in Khartoum state; the study participants were only nurses and midwives. 20. The finding of this study related to attitude 40.3% respondants responds the questions correctly; this finding is consistent with the study conducted in Bamenda Health District, NWR, Cameroon, 44%. This may be a result of the study participant similarity [16]. However, unlike this study, the study was conducted in Saudi and Saad Abul-Ella hospitals in Khartoum; state occupation, educational degree, and work experience were not significantly associated with knowledge of HBV infection [17]. This is maybe a result of the study participant difference; in this study, almost all health care categories were incorporated in a study in contrast Saad Abul-Ella hospitals in Khartoum state study participants were only nurses and midwives. This study showed that radiographers (66%) have answered correctly attitude questions followed by medical doctors (58%) and pharmacists (54.8), respectively. Of 98 (23.2%), the study participants had no concern being infected with HBV, and of 143 (33.6%), participants had a belief that changing gloves is a waste of time. In contrast, 274 (64.9%) of the participants have believed the HBV vaccine is safe and effective. Of 307 (72.7%), study participants believed that following infection control guidelines would protect them from HBV infection, but only 31.3% of participants believed that postexposure prophylaxis could prevent from HBV infection. This study finding related to attitude 40.3% of study participants responds the questions correctly. This finding is consistent with the study that was conducted in Bamenda Health District, NWR, Cameroon, 44.%. This may be a result of the study participant similarity [16]. This is may be as a result of in both study majority of the participants were nurse professionals. This study was lower than the study that was done in Suntreso Government Hospital, Ghana 69.14-91.9%, and in Saudi and Saad Abul-Ella hospitals in Khartoum state (86.4%) showed a positive attitude towards HBV [17, 18], respectively. This is maybe as a result of the numbers of the respondent difference between them. In this study, educational status was significantly associated with the attitude level of HBV.

This study showed that medical laboratory professionals (70%) answered correctly with practice questions followed by pharmacy professionals (68.7%), psychiatrists (50%), radiographers (33.3), and medical doctors (32.2%), respectively. In this study of 158 (37.4%), study participants were screened for HBV; however, the majority of 255 (60.4%) health care workers had vaccinated against HBV which is lower than the study was done in Mogadishu, Somalia (83.7%); in this study finding related to screeing for HBV and vaccination against the infection were 86.3% [12]. This is may be as a result of shortage of screening kits and vaccination in this study area.

About 410 (97.2%) of the respondents did wear gloves, but 191 (45.3%) of the respondents changed gloves for each patient. Of 192 (45.5%), the participants had a needle stick injury. Of 274 (64.9%), the participants disposed of sharps properly.

From this study, the average good practice level was 34.8% which is lower than in the Ho Municipality of the Volta Region of Ghana; 60% have undergone screening for hepatitis B virus [8], and the study was conducted in Saudi and Saad Abul-Ella hospitals in Khartoum state, Sudan; 65.5% of the respondents had a safe practice [17]. This is maybe a result of difference in training regarding HBV infection for the professionals among different study areas. Based on our finding, most of study participant vaccination status showed that vaccinated against HBV is 255 (60.4%). This funding was higher than the study was done in the Ho Municipality of the Volta Region of Ghana 49.4% have taken the full three doses of vaccines [8]. This is may be due to shortage of vaccine supply, or participants may be were not take vaccine before study period.

Regarding needle stick injury, this finding shows that 192 (45.5%) had a needle stick injury which was higher than the study that was done in Bantama, Ghana; among health care workers, 29.1% had needle stick injury [18]. However, this study is lower than the study that was conducted in four public hospitals in Wad Medani, Sudan; 81.0% of the participated HCWs were exposed to accidental needle stick injury [19]. This is maybe a result of differences in waste management habits and educational status differences between the participants. This study showed that the multivariate analysis of the practice levels was professions and educational status of the participants had shown overall significant effect on a good practice.

## 5. Conclusion and Recommendation

In conclusion, this study showed that most of the health care professionals in Tibebe Ghion Specialized Hospital have poor knowledge, negative attitude, and malpractice towards HBV infection. Therefore, continual professional training programs on HBV infection include increased vaccination coverage rate and postexposure prophylaxis of health care workers especially for highly exposed professionals. Ministry of Health should collaborate with the health bureau for continual professional training about HBV mode of transmission and preventive measures as well as providing personal protective equipment and making them be upgrading their profession because improving educational status has their own role to have good knowledge, positive attitude, and good practice to prevent of HBV infection.

## 6. Limitations of the Study

Overall, the study was conducted by health care professionals at Tibebe Ghion Specialized Hospital. Therefore, it might not be representative of all health professionals across Ethiopia. Another limitation was that measurements for the level of knowledge, attitude, and practice were taken from each primary study, and operational definitions may have differed between the studies. The studies were included only health professionals but did not include nonhealth professionals working in the hospital. In addition, this study was cross-sectional in nature, so it could not show cause and effect relationship; it needs cohort study.

## Figures and Tables

**Table 1:** Sociodemographic characteristics of the respondent at Tibebe Ghion Specialized Hospital from January 1 to February 30, 2021.

Variables		Frequency (*N*)	Percent
Sex	Male	243	57.6%
	Female	179	42.4%
Age	18-25	85	20.1%
	26-33	308	73%
	34-41	29	6.9%
Marital status	Single	179	42.4%
	Married	228	54%
	Separated	13	3.1%
	Divorced	2	0.5%
Professions	M. doctor	93	22%
	M. lab.	20	4.7%
	Nurse	210	49.8%
	Midwife	48	11.4%
	Pharmacy	32	7.6%
	Others^∗^	19	3.5%
Educational status	Diploma	66	15.6%
	Degree	271	64.2%
	MSc	59	14.0%
	Specialist	26	6.2%
Work experience	<2years	98	23.2%
	>2years	324	76.8%

**Table 2:** Knowledge-related questions about HBV early detection, transmission, and prevention.

Knowledge questions	Yes, *N* (%)	No, *N* (%)	Not sure, *N* (%)
Can HBV causes liver cancer	359 (85.1)	19 (4.5)	44 (10.4)
Can HBV carrier transmit the infection	351 (83.2)	19 (4.5)	52 (12.3)
Can HBV spread by casual hand shaking	196 (46.4)	162 (38.4)	64 (15.2)
Can HBV spread by contact with open wound	403 (95.5)	11 (2.6)	8 (1.9)
Can HBV transmitted by contaminated blood and body fluids	410 (97.2)	6 (1.4)	5 (1.2)
Can HBV transmitted by unsterilized syringes, needles, and surgical instruments	382 (90.5)	12 (2.8)	28 (6.6)
Do you think HBV transmitted by unsafe sex	296 (70.1)	37 (8.8)	89 (21.1)
Can HBV transmitted with contaminated water	145 (34.4)	224 (53.1)	53 (12.6)
HBV is 50 to 100 times more infectious than HIV	157 (37.2)	87 (20.6)	178 (42.2)
Do you think vaccine can prevent HBV infection	350 (82.9)	8 (1.9)	64 (15.2)
Do you think HBV has laboratory tests	375 (88.9)	15 (3.6)	32 (7.6)
Do you know HBV has postexposure prophylaxis	174 (41.2)	111 (26.3)	137 (32.5)
Can HBV cured/treat	90 (21.3)	227 (53.8)	105 (24.9)

**Table 3:** Knowledge level of the health professionals about HBV early detection, transmission, and prevention at Tibebe Ghion Specialized Hospital from January 1 to February 30, 2021.

Professions	Knowledge level		
	Poor, *N* (%)	Good, *N* (%)	Total
Medical doctors	8 (8.6)	85 (91.4)	93
Medical laboratory	4 (20)	16 (80)	20
Nurses	97 (46.2)	113 (53.8)	210
Midwives	32 (66.7)	26 (33.3)	48
Pharmacy	5 (15.6)	27 (84.4)	32
Anesthesia	5 (83.3)	1 (12.7)	6
Radiographer	1 (33.3)	2 (66.7)	3
Psychiatrist	1 (12.7)	5 (83.3)	6
Dentist	2 (100)	0	2
Ophthalmologist	0	2 (100)	2

**Table 4:** Attitude-related questions about HBV early detection, transmission, and prevention.

Attitude questions	Agree, *N* (%)	Disagree, *N* (%)	Not sure, *N* (%)
No concern of being infected with HBV	98 (23.2)	312 (73.9)	10 (2.4)
HBV vaccine is safe and effective	274 (64.9)	64 (15.2)	84 (19.9)
Change of glove during blood collection and testing is waste of time	142 (33.6)	276 (65.4)	4 (0.9)
All patients should be tested for HBV before receive health care	228 (54.0)	161 (38.2)	33 (7.8)
I do not feel comfortable to take care of people with HBV	215 (50.9)	182 (43.1)	25 (5.9)
Following infection control guidelines will protect from being infected with HBV at work	307 (72.7)	64 (15.20)	51 (12.1)
Postexposure prophylactic can prevent from HBV infection	132 (31.3)	83 (19.7)	206 (48.8)

**Table 5:** Attitude levels of the health professionals about HBV early detection, transmission, and prevention at Tibebe Ghion Specialized Hospital from January 1 to February 30, 2021.

Professions	Attitude level		
	Negative, *N* (%)	Positive, *N* (%)	Total
Medical doctors	39 (41.9)	54 (58.1)	93
Medical laboratory	13 (65)	7 (35)	20
Nurses	147 (70)	63 (30)	210
Midwives	32 (66.7)	16 (33.3)	48
Pharmacy	10 (45.5)	22 (54.5)	32
Anesthesia	4 (66.7)	2 (33.3)	6
Radiographer	1 (33.3)	2 (66.7)	3
Psychiatrist	4 (66.7)	2 (33.3)	6
Dentist	1 (50)	1 (50)	2
Ophthalmologist	1 (50)	1 (50)	2

**Table 6:** Practice-related questions about HBV early detection, transmission, and prevention.

Practice questions	Yes, *N* (%)			No, *N* (%)
Have you ever screened from HBV	158 (37.4)			264 (62.6)
Have got vaccine against HBV	255 (60.4)			167 (39.6)
How many dose of HBV vaccine did you receive	1 dose	2 doses	3 doses	
	4 (.9)	31 (7.3)	220 (52.1)	
Do you wear gloves when carrying out procedures	410 (97.2)			12 (2.8)
I always change glove for each patient during blood taking	191 (45.3)			231 (54.7)
Have you ever hand a needle stick injury	192 (45.5)			230 (54.5)
I always report for needle stick injury	145 (34.4)			277 (65.6)
Do you dispose of sharps properly after a procedure	274 (64.9)			148 (35.1)

**Table 7:** Practice levels of the health care professionals about HBV early detection, transmission, and prevention at Tibebe Ghion Specialized Hospital from January 1 to February 30, 2021.

Professions	Practice level		
	Malpractice, *N* (%)	Good practice, *N* (%)	Total
Medical doctors	63 (67.7)	30 (32.3)	93
Medical laboratory	6 (30)	14 (70)	20
Nurses	148 (70.5)	62 (29.5)	210
Midwives	34 (70.8)	14 (29.2)	48
Pharmacy	10 (31.3)	22 (68.7)	32
Anesthesia	5 (83.3)	1 (16.7)	6
Radiographer	2 (66.7)	1 (33.3)	3
Psychiatrist	3 (50)	3 (50)	6
Dentist	2 (100)	0	2
Ophthalmologist	2 (100)	0	2

**Table 8:** Bivariate and multivariate analyses of factor associated with good knowledge towards HBV infections.

Variables		Knowledge		COR (95% CI)	*P* value	AOR (95% CI)	*P* value
		Poor	Good				
Sex	Male	67 (27.6)	176 (72.6%)	2.03 (1.35, 3.05)	0.001	1.3 (0.79, 2.12)	0.3
	Female	78 (43.6)	101 (56.4%)	Ref		Ref	
Profession	M. doctor	8 (8.6)	85 (91.4%)	Ref		Ref	
	M. laboratory	4 (20)	16 (80%)	0.38 (0.10, 1.4)	0.14	0.81 (0.21, 3.19)	0.81
	Nurse	97 (46.2)	113 (53.8%)	0.11 (0.05, 0.24)	<0.001	0.17 (0.07, 0.38)	<0.001
	Midwife	22 (45.8)	26 (54.2%)	0.11 (0.04, 0.28)	<0.001	0.19 (0.07, 0.5)	0.001
	Pharmacy	5 (15.6)	27 (84.4%)	0.51 (0.15, 1.69)	0.26	0.72 (0.21, 2.51)	0.1
	Anesthesia	5 (83.3)	1 (6.7%)	0.02 (0.002, 0.18)	0.001	0.02 (0.002, 0.23)	0.001
	Radiograph er	1 (33.3)	2 (66.7%)	0.19 (0.02, 2.31)	0.19	0.4 (0.03, 5.71)	0.5
	Psychiatrist	1 (16.7)	5 (83.3%)	0.47 (0.05, 4.53)	0.5	0.6 (0.06, 5.71)	0.6
Education al status	Diploma	36 (54.5%)	30 (45.5%)	Ref		Ref	
	Degree	89 (32.8%)	182 (67.2%)	2.45 (1.42,4.24)	0.001	1.89 (1.014, 3.53)	0.045
	MSc	20 (33.9)	39 (66.1%)	2.34 (1.13, 4.83)	0.02	1.97 (0.87, 4.46)	0.1
Work experience	<2years	51 (52%)	47 (48%)	Ref		Ref	
	>2years	94 (29%)	230 (71%)	2.66 (1.67, 4.22)	<0.001	2.37 (1.38, 4.07)	0.002

**Table 9:** Bivariate and multivariate analyses of factors associated with attitude levels off HBV infection.

Variables		Attitude		COR (95% CI)	*P* value	AOR (95% CI)	*P* value
		Negative	Positive				
Sex	Male	127 (52.3%)	116 (47.7)	2.11 (1.42, 3.18)	<0.001	1.33 (0.83, 2.12)	0.24
	Female	125 (69.8%)	54 (30.2%)		Ref		Ref
Profession	M. doctor	39 (41.9)	54 (58.1)		Ref		Ref
	M. lab	13 (65)	7 (35)	0.39 (0.14, 1.06)	0.07	0.67 (0.23, 1.99)	0.47
	Nurse	147 (70)	63 (30)	0.31 (0.19, 0.51)	<0.001	0.46 (0.25, 0.83)	0.1
	Midwives	32 (66.7)	16 (33.3)	0.36 (0.17, 0.75)	0.006	0.61 (0.27, 1.37)	0.23
	Pharmacy	10 (31.3)	22 (68.7)	1.59 (0.68, 3.73)	0.3	2.4 (0.94, 6.05)	0.7
	Anesthesia	4 (66.7)	2 (33.3)	0.36 (0.06, 2.07)	0.25	0.42 (0.07, 2.47)	0.33
	Radiographer	1 (33.3)	2 (66.7)	1.44 (0.13, 16.4)	0.77	3.4 (0.27, 43.8)	0.34
	Psychiatrist	4 (66.7)	2 (33.3)	0.72 (0.04, 11.96)	0.82	0.4 (0.07, 2.4)	0.31
Educational status	Diploma	52 (78.8)	14 (21.2)		Ref		Ref
	Degree	163 (60.1)	108 (39.9)	2.5 (0.126, 4.66)	0.006	2.49 (1.23, 5.02)	0.01
	MSc	32 (54.2)	27 (45.8)	3.1 (1.44, 6.85)	0.004	3.97 (1.69, 9.28)	0.001
	Specialist	5 (19.2)	21 (80.8)	15.6 (4.99, 48.78)	<0.001	9.78 (2.69, 35.5)	0.001

**Table 10:** Bivariate and multivariate analyses of associated factors of practice levels towards HBV infection.

Variables		Practice level		COR (95% CI)	*P* value	AOR (95% CI)	*P* value
		Malpractice	Good practice				
Sex	Male	143 (58.8)	100 (41.2)	1.96 (1.29, 2.99)	0.002	1.54 (0.93, 2.53)	0.09
	Female	132 (73.7)	47 (26.3)		Ref		Ref
Profession	M. doctor	63 (67.7)	30 (32.3)		Ref		Ref
	M. lab	6 (30)	14 (70)	4.9 (1.71, 14.01)	0.003	17.42 (5.02, 60.5)	<0.001
	Nurse	148 (70.5)	62 (29.5)	0.89 (0.52, 1.49)	0.6	2.12 (1.025, 4.38)	0.04
	Midwife	34 (70.8)	14 (29.2)	0.87 (0.405, 1.85)	0.7	2.48 (0.98, 6.29)	0.05
	Pharmacy	10 (31.3)	22 (68.7)	4.62 (1.95, 10.97)	0.001	11.2 (4.02, 31.42)	<0.001
Educational status	Diploma	54 (81.8)	12 (18.2)		Ref		Ref
	Degree	182 (67.2)	89 (32.8)	2.2 (1.12, 4.32)	0.02	3.4 (1.58, 7.35)	0.002
	MSc	31 (53.4)	28 (46.6)	4.06 (1.81, 9.12)	0.001	5.3 (2.12, 13.11)	<0.001
	Specialist	8 (33.3)	18 (69.2)	10.13 (3.57, 28.69)	<0.001	31.4 (8.59, 114.5)	<0.001

## Data Availability

Most of the data generated or analyzed during this study are included in this article. Additional data will be made available upon request to the primary/corresponding author.

## Abbreviations

Ab: Antibody
ALT: Alanine transaminase
AOR: Adjusted odds ratio
CI: Confidence interval
COR: Crude odd ratio
DNA: Doxy ribonucleic acid
HBcAg: Hepatitis B core antigen
HBeAg: Hepatitis B envelop antigen
HBsAb: Hepatitis B surface antibody
HBsAg: Hepatitis B surface antigen
HBV: Hepatitis B virus
HCWs: Health care workers
IGM: Immunoglobulin meu
KAP: Knowledge, attitude and practice
NTC: Nurses training college
TGSH: Tibebe Ghion Specialized Hospital
WHO: World Health Organization.
